# Deep learning-based risk stratification of preoperative breast biopsies using digital whole slide images

**DOI:** 10.1186/s13058-024-01840-7

**Published:** 2024-06-03

**Authors:** Constance Boissin, Yinxi Wang, Abhinav Sharma, Philippe Weitz, Emelie Karlsson, Stephanie Robertson, Johan Hartman, Mattias Rantalainen

**Affiliations:** 1 Department of Medical Epidemiology and Biostatistics, Karolinska Institutet, Stockholm, Sweden; 2 Department of Oncology-Pathology, Karolinska Institutet, Stockholm, Sweden; 3 Department of Clinical Pathology and Cancer Diagnostics, Karolinska University Hospital, Stockholm, Sweden; 4 MedTechLabs, BioClinicum, Karolinska University Hospital, Stockholm, Sweden

**Keywords:** Breast biopsies, Grade, Artificial intelligence

## Abstract

**Background:**

Nottingham histological grade (NHG) is a well established prognostic factor in breast cancer histopathology but has a high inter-assessor variability with many tumours being classified as intermediate grade, NHG2. Here, we evaluate if DeepGrade, a previously developed model for risk stratification of resected tumour specimens, could be applied to risk-stratify tumour biopsy specimens.

**Methods:**

A total of 11,955,755 tiles from 1169 whole slide images of preoperative biopsies from 896 patients diagnosed with breast cancer in Stockholm, Sweden, were included. DeepGrade, a deep convolutional neural network model, was applied for the prediction of low- and high-risk tumours. It was evaluated against clinically assigned grades NHG1 and NHG3 on the biopsy specimen but also against the grades assigned to the corresponding resection specimen using area under the operating curve (AUC). The prognostic value of the DeepGrade model in the biopsy setting was evaluated using time-to-event analysis.

**Results:**

Based on preoperative biopsy images, the DeepGrade model predicted resected tumour cases of clinical grades NHG1 and NHG3 with an AUC of 0.908 (95% CI: 0.88; 0.93). Furthermore, out of the 432 resected clinically-assigned NHG2 tumours, 281 (65%) were classified as DeepGrade-low and 151 (35%) as DeepGrade-high. Using a multivariable Cox proportional hazards model the hazard ratio between DeepGrade low- and high-risk groups was estimated as 2.01 (95% CI: 1.06; 3.79).

**Conclusions:**

DeepGrade provided prediction of tumour grades NHG1 and NHG3 on the resection specimen using only the biopsy specimen. The results demonstrate that the DeepGrade model can provide decision support to identify high-risk tumours based on preoperative biopsies, thus improving early treatment decisions.

**Supplementary Information:**

The online version contains supplementary material available at 10.1186/s13058-024-01840-7.

## Background

Breast cancer is currently the most common cancer type globally [1]. In the majority of cases, suspicious breast lesions are initially identified by mammography screening, which is recommended in most developed countries for early detection of breast cancer [2, 3]. For women with a suspicious lesion, a preoperative core needle biopsy is performed to histologically assess the breast tissue [4]. Evaluation of the biopsy by pathologists is key to diagnose breast cancer, where morphological information and biomarker analysis are paramount to guide further surgical and oncological therapy decisions [5].

Tumour grading is a cornerstone in the histopathological assessment of breast cancer, not only in the resected tumour specimen but it is also of importance in the biopsy specimen [6]. Histological grade reflects the degree of differentiation of a tumour by comparing the similarity of malignant cells to that of normal breast terminal duct lobular units [7]. Currently, the most commonly used grading method is the Nottingham Histological Grade (NHG) adapted by Elston-Ellis following work from Bloom-Richardson [8, 9]. Histological grading relies on the performance and expertise of pathologists, and evaluates three morphological features: the degree of tubular formation (gland architecture), nuclear pleomorphism (nucleus size and shape) and the mitotic count [9]. Each of these three morphological features is given a score from 1 to 3 by the pathologist and are then combined to obtain the final NHG grade on a score from 1 to 3. Histological grade is an important prognostic feature of breast cancer, with NHG1 having a good prognosis and NHG3 tumours being associated with poor prognosis, independently of the morphological subtype and nodal status [10–12]. Therefore, tumour grade plays an important role in guiding treatment decisions [13]. However, about 50% of all resected breast cancer specimens are diagnosed as NHG2, which has limited clinical value for treatment decisions [14–16]. One of the major challenges with histological grading is that it relies on the experience, expertise and interpretation of the pathologist, and high inter-observer and inter-laboratory variabilities are well described [7, 14].

Histological grade assessment is even more complicated in biopsy specimens with very limited tumour material and frequent tissue artefacts [14]. This causes significant discrepancies between the biopsy grading and the histological grade assigned on the surgically resected specimen [17]. These uncertainties are accompanied by the fact that a greater number of biopsy samples are not even assigned a grade, and that up to 70% of the biopsy samples are assigned the intermediate grade, NHG2 [15, 18, 19].

The recent advances in computational pathology based on the availability of large amounts of digitised whole-slide histopathological images, as well as the development of novel artificial intelligence technologies, has enabled model-based grading of tumours in resected specimens [20–22]. Wang et al. have also shown by developing the DeepGrade model using resected NHG1 and NHG3 tumours that this technology enabled further risk stratification of intermediate-risk NHG2 patients into two risk subgroups with independent prognostic value [23]. Risk stratification is particularly relevant in patients with oestrogen receptor (ER)-positive/human epidermal growth factor receptor 2 (HER2)-negative tumours, since high-risk (NHG3) patients will typically be provided chemotherapy in addition to endocrine therapy, and low-risk (NHG1) patients would be spared chemotherapy in order to avoid overtreatment, whereas the intermediate NHG2 group is uninformative and has limited clinical value for treatment decisions.

In this study, we aim to assess if the DeepGrade model [23], developed using resected tumour specimens, could be applied to risk-stratify tumours using only the biopsy specimens. This would allow earlier identification of high-risk tumours from the initial biopsy specimens, and further improve information that can be used in the treatment planning at the preoperative stage.

## Methods

### Patients

**Fig. 1:**
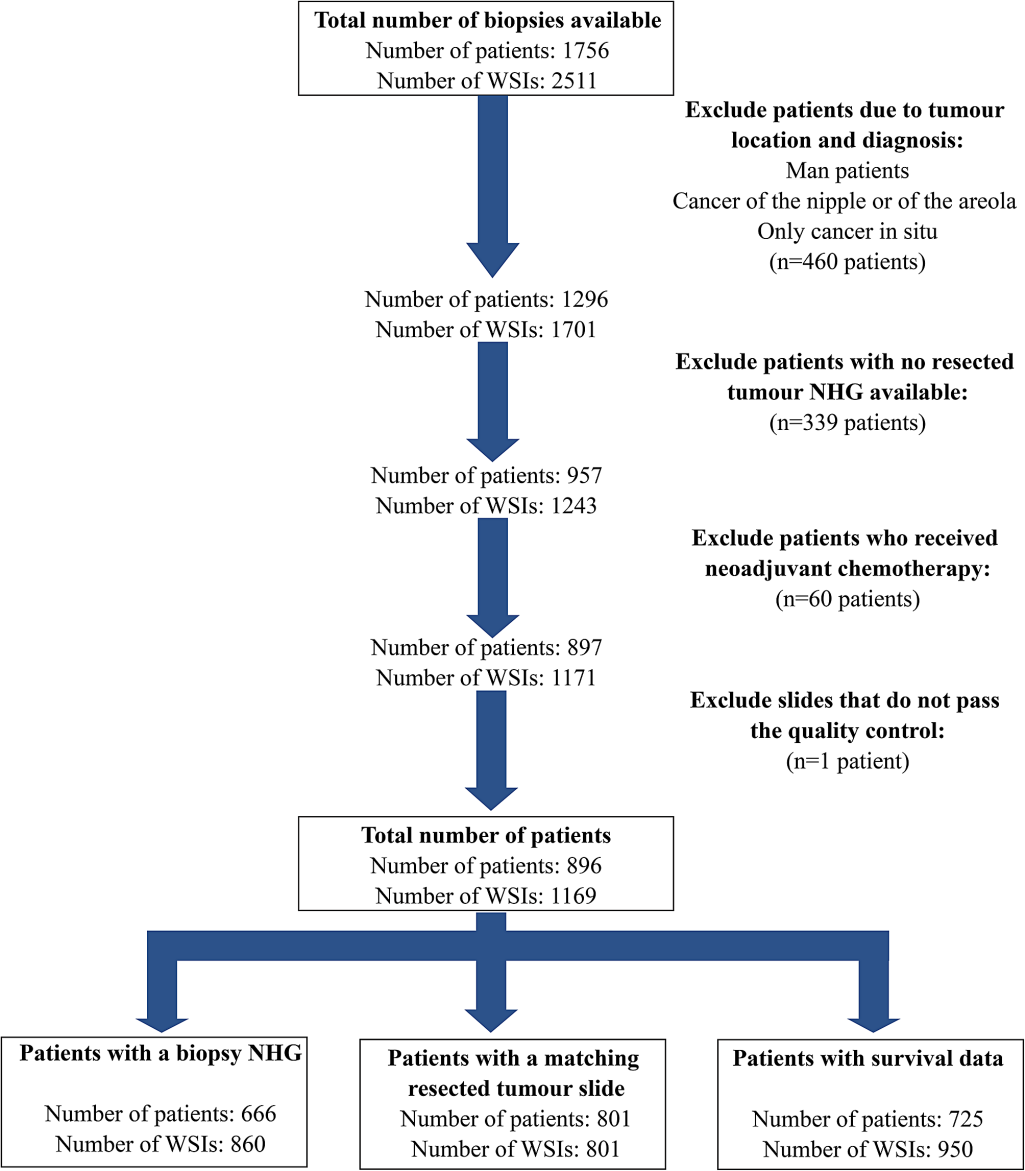
CONSORT diagram. The data used contained whole slide images (WSI) of biopsies for 896 patients who had a resected tumour Nottingham Histological Grade (NHG) and who did not receive neoadjuvant chemotherapy. Out of these, a total of 666 patients had a biopsy NHG, 801 patients had a matching resected tumour slide, on which DeepGrade predictions could also be performed, and, survival data was available for 725 patients. A total of 525 patients (682 WSI) are in all three subanalyses

This retrospective study included female patients who underwent a breast biopsy at the Stockholm South General Hospital in Stockholm, Sweden between June 2012 and May 2018. Patients diagnosed with invasive breast cancer as their primary diagnosis and who had undergone a surgical removal of their tumour within two months following their biopsy without receiving neoadjuvant therapy were included in the study. See Fig. 1 for detailed explanation of the selection criteria. A total of 1169 whole slide images (WSI) from 896 patients were included in the final analyses. The WSI from the resected tumour specimens of 801 of these patients were also available and used for comparison of the prediction of DeepGrade risk group on this material. Clinical data was retrieved retrospectively from the Swedish National Breast Cancer (NKBC) Registry as well as from the patient’s pathology reports when data on the NHG status was not available in the registry. The NKBC registry includes data from newly diagnosed patients with primary in-situ or invasive tumours in Sweden and covers both a full pathology report as well as survival based on follow-up routines [24]. The patients’ NHG were assessed as part of routine clinical care, and separately for the biopsy and resected tumour specimens. This study was reviewed and approved by the Swedish Ethical Review Authority.

### WSI and deep learning model

For each patient between one and seven haematoxylin and eosin (H&E) stained formalin-fixed paraffin-embedded (FFPE) histopathology slides of biopsy specimens were digitised in-house using either Hamamatsu Nanozoomer XR or Hamamatsu Nanozoomer S360 scanners (Hamamatsu Photonics K.K., Shizuoka, Japan) at 40X magnification (0.227 μm/pixel and 0.230 μm/pixel, respectively). Methodology for pre-processing of the WSI was performed according to the methodology previously described [23]. Initially, tissue segmentation was performed by transforming lower-level representations extracted from the WSI’s resolution pyramids obtained using OpenSlide [25]. These were then transformed from RGB to HSV colour space. Two masks were then generated for each slide, one for filtering out hue values lower than 0.75, the other adding a maximum value of 25 to the Otsu’s threshold [26] in order to remove non-tissue areas while reducing the removal of the tissue regions due to the high threshold value on the transformed saturation channel in some cases.

WSI regions included in the tissue mask were tiled into image tiles of 598 × 598 pixels with a down-sampled resolution equivalent to 20 × (271 μm x 271 μm). Due to the small tissue area in biopsy specimens, tiling was performed with 75% overlap on both the vertical and horizontal axes between two consecutive tiles. Next, in order to ensure quality of the data, remove unsharp tiles, any remaining tiles with background, those with adipose tissue, and blurred tiles were all excluded by measuring a variance of the Laplacian filter and excluding the tiles with a value lower than 500 [23]. Lastly, to address the stain variabilities in WSI, colour normalisation across each WSI was performed using the method described by Macenko et al [27], and as implemented by Wang et al [23]. Colour normalisation was applied with the same factor to all tiles within a WSI. Using reference stain vectors [28] and slide level stain vectors obtained using 100 randomly selected tiles per slide, colour normalisation could be applied to each tile towards the reference stain vectors. For the 801 patients with preoperative biopsies and a matching resected tumour WSI, a similar pre-processing method was performed for the WSI pre-processing with two significant changes. First, no overlap between two consecutive tiles was considered. Secondly, after the colour normalisation step, a tumour segmentation model previously developed [23] was applied to include only the tiles from the invasive cancer regions in the resected specimens for further downstream analysis. After pre-processing, a total of 11,955,755 tiles were used for predictions from biopsy specimens, and 1,157,871 were used from the surgical resection specimens.

### Histological grade prediction

Prediction of low- (NHG1) and high- (NHG3) risk tumours on the biopsy WSI was performed using an ensemble of 20 convolutional neural network (CNN) models previously developed as the DeepGrade model [23]. The DeepGrade models were trained to classify NHG1 and NHG3 tumours in WSI from resected specimens. Each model uses Inception V3 model pre-trained with ImageNet [29] as the base model. The Inception V3 model consists of a stem block constituted of four 3 × 3 convolutional layers and one 1 × 1 convolutional layer as well as two max pooling layers. It then employs three inception blocks (A, B and C) each consisting of 1 × 1, 3 × 3 and 5 × 5 convolutional filters together with regularization. Inception blocks A and B also include an average pooling layer while block C includes a max pooling layer. Block A is followed by a reduction block that includes further convolutional layers and one max pooling layer while block B employs 3 × 3 convolutions with strides to downsample the feature maps. Finally, there is an auxiliary classifier block to prevent vanishing gradient. A fully connected layer of 1024 hidden units and Rectified Linear Unit (RELU) activation function were added before the final layer. Stochastic gradient descent was used to update parameters from all layers with an adaptive learning rate starting from 10^− 3^ but reduced by 50% each time the model performance stopped improving for 10 epochs. Cross-entropy loss was used for the binary outcome NHG1 versus NHG3. The initial 20 CNN models were trained on 844 WSI, of which 173 patients’ biopsy WSI were also included in this study. Each model in DeepGrade outputs the two class prediction probabilities for each tile (P(NHG_3_|tile_i_) and P(NHG_1_|tile_i_)). The P(NHG_3_|tile_i_) class probability from each of the 20 models in the ensemble were averaged to provide the tile-level prediction. In order to obtain the patient-level predictions, all the tile-level predictions of all the WSI from each patient and the upper-percentile (99%) of the tile level predictions were considered. Regarding resected tumour specimens, as tumour detection was previously performed, a lower threshold was used with the upper-quartile (75%) of the tile level predictions being considered. For NHG1 and NHG3, prediction performance of the DeepGrade model was evaluated against clinically assigned NHG by pathologists on both the biopsy specimen (biopsy NHG) and on the surgically resected specimen (resected tumour NHG). The prediction performances on the patient levels were measured using the receiver operating characteristic (ROC) curves and the linked area under the curve (AUC) using R package pROC [30]. The most optimal threshold for binary assignment into low- and high-risk groups was then determined using the Youden’s J statistic [31] compared to the resected tumour grade. A separate threshold was calculated for DeepGrade predictions on resected tumour specimens. Agreement between the assigned NHG (from the biopsy specimen or the resected tumour specimen) and the obtained DeepGrade risk group was measured using Cohen’s kappa and the following interpretations: 0-0.20: slight agreement, 0.21–0.40: fair agreement, 0.41–0.60: moderate agreement, 0.61–0.80: substantial agreement and 0.81-1.00: almost perfect agreement [32, 33]. Sensitivity or recall was measured as the probability of DeepGrade-high when the patient had NHG3, while specificity was measured as the probability of DeepGrade-low when the patient had NHG1. Furthermore, the DeepGrade model was applied on NHG2 tumours to sub-stratify the tumours into two groups: low- and high-risk groups. The classification performance of the DeepGrade on the biopsy WSI was also compared to the classification performed on the resection WSI.

### Survival analyses

Finally, the rates of recurrence-free survival (RFS) as defined by the presence of a locoregional or distant recurrence or death were compared between patients who were assigned in the DeepGrade-high and DeepGrade-low groups. The time-to-event was defined as the number of days between the date of initial diagnosis and either date of recurrence or loss of follow-up. The R packages ‘survival’ and ‘survminer’ were used to visualise the survival outcomes between groups, and the ‘forestmodel’ package was used to estimate adjusted hazard ratios (HRs) using multivariate Cox proportional hazards regression models. The other risk factor used in the model was age, considered as the only factor available at time of biopsy. Further sub-analyses were performed on oestrogen receptor (ER)-positive and human epidermal growth factor receptor 2 (HER2)-negative cases as determined by immunohistochemical and/or in situ hybridisation staining and available in the pathology report.

## Results

### Agreement between clinical grades on biopsy and resected specimens

**Fig. 2:**
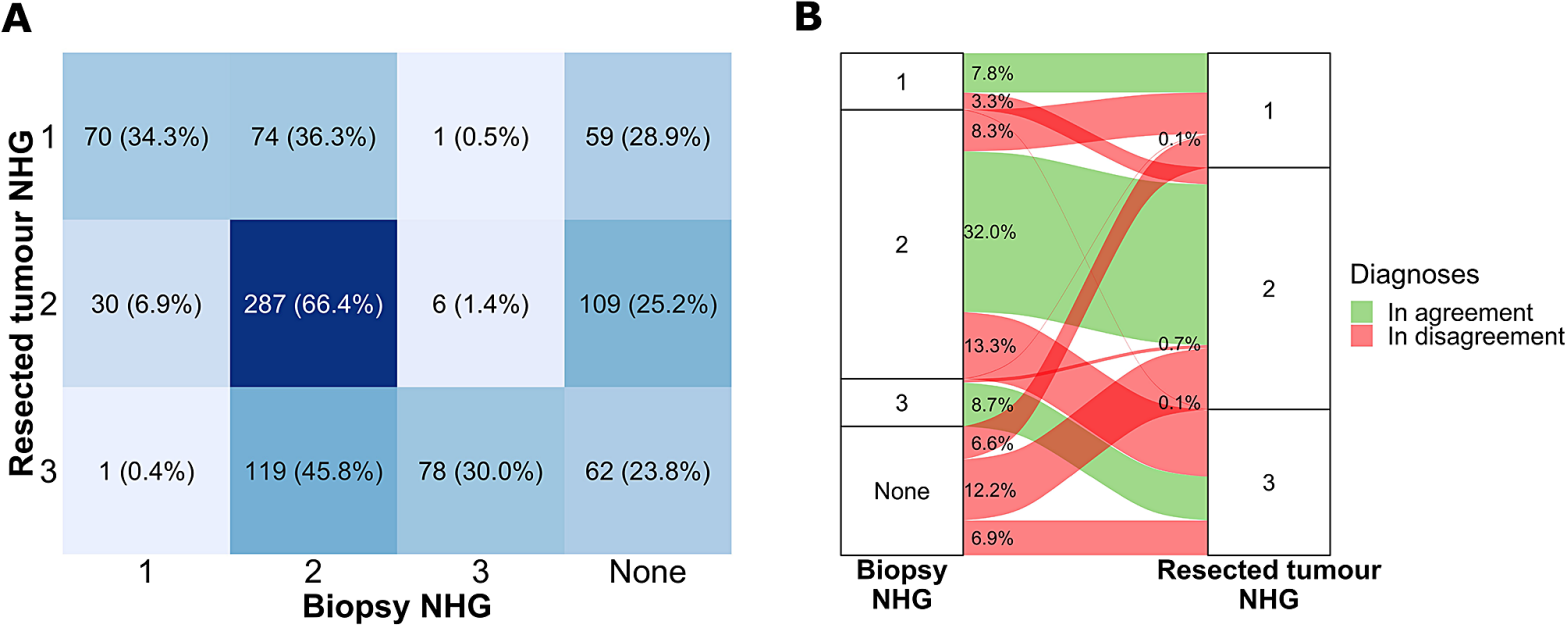
Comparison between clinical Notthingham Histological Grade (NHG) assigned by pathologists on the biopsy and surgically resected specimens. **A.** confusion matrix, **B.** Sankey plot, diagnoses were in agreement when the same NHG was assigned to the biopsy specimen and to the resected tumour specimen

First, we assessed the discrepancies between the clinical assignment of NHG on the biopsy and subsequent resected specimens (Fig. 2). A quarter of the patients did not have a NHG assigned on the biopsy specimen in clinical routine, and 72% of the patients who had a biopsy NHG available were of NHG2. The overall agreement between the clinical grade assignments on the biopsy and on the resected tumour specimen was 65.5% when including patients for whom we had both diagnoses. When considering only cases that had a resected tumour NHG1 or NHG3, less than a third (148 out of 463 cases) also had a NHG1 or NHG3 in the biopsy specimen, the rest being assigned NHG2 or not having a grade at all. We observed a fair agreement with the Cohen’s kappa value of 0.40 (95% CIs: 0.34;0.46) between specimen types.

### Assessment of the DeepGrade classification performance on the biopsy specimen

**Fig. 3:**
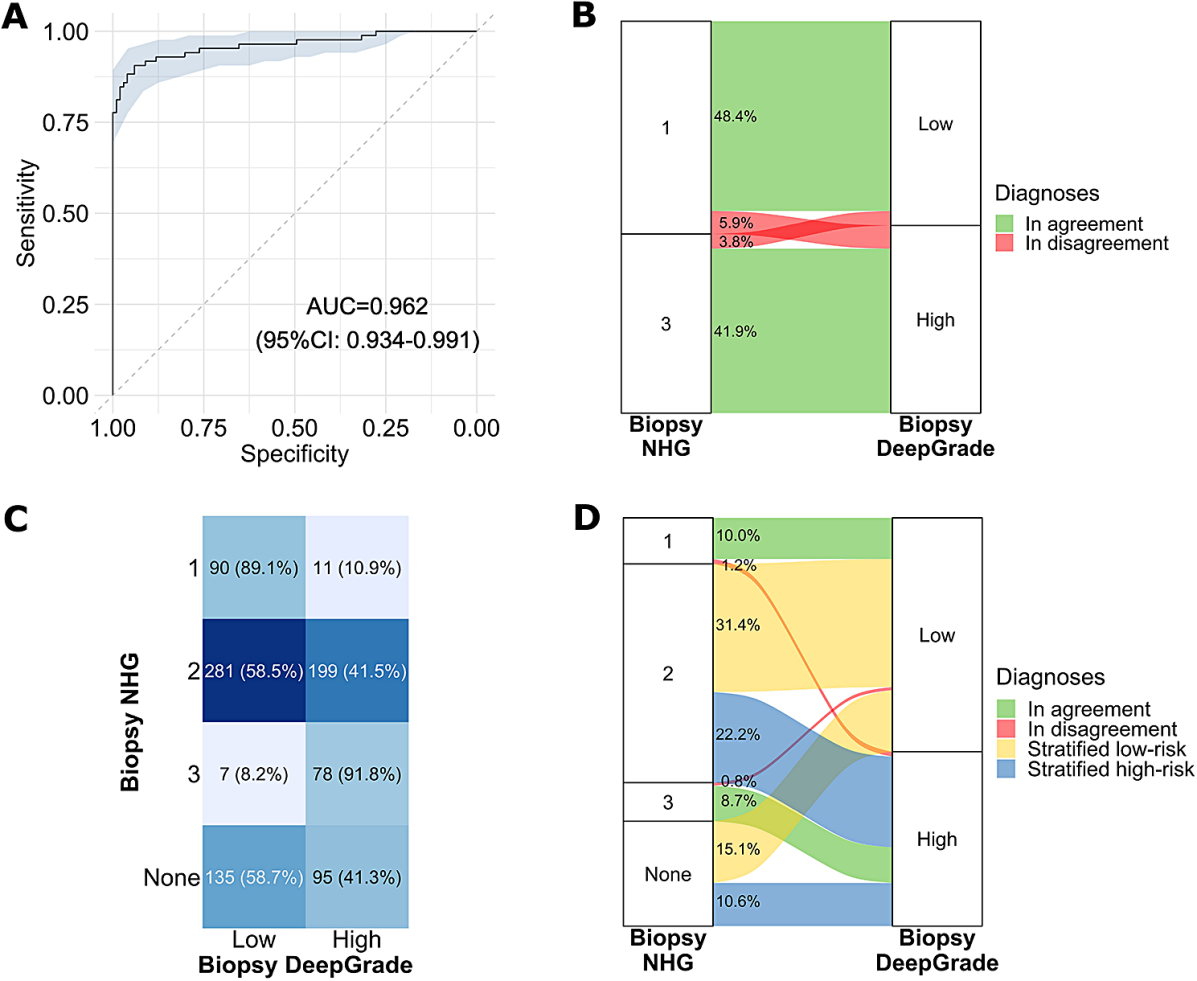
DeepGrade prediction results obtained on biopsy specimens compared to the clinical biopsy Nottingham Histological Grade (NHG) assigned by pathologists. **A.** Receiver Operating Curve (ROC) of the patient-level predictions obtained by the DeepGrade model compared to biopsy NHG1 and NHG3. **B.** Sankey plot of the biopsy NHG compared to the obtained DeepGrade risk group for the 186 patients who had a biopsy NHG1 or NHG3. **C.** Confusion matrix for all 896 patients comparing biopsy NHG and predicted DeepGrade risk group. **D.** Sankey plot for all patients. Diagnoses were in agreement when patients were DeepGrade-low and NHG1 or DeepGrade-high and NHG3. Patients with biopsy NHG2 or with no biopsy NHG were stratified as either low-risk or high-risk

We evaluated the risk classification performance of the DeepGrade model on the biopsy specimens. We observed an AUC score of 0.962 (95% CI: 0.934; 0.991) for DeepGrade predictions compared to biopsy NHG1 and NHG3 (Fig. 3A). For 168 out of 186 patients (90.3%) with biopsy NHG1 or NHG3 (Fig. 3B-C) the DeepGrade model and the pathologists were in agreement, representing an almost perfect agreement with a kappa value of 0.81. Sensitivity was of 91.7% while specificity was of 89.1%. Out of all 896 patients, only 0.8% (7) biopsy NHG3 tumours were assigned to the DeepGrade low-risk group (Fig. 3C-D). Out of the 230 patients without a biopsy NHG, 135 (58.7%) were classified in the DeepGrade low-risk group and 95 (41.3%) were classified in the high-risk group (Fig. 3C-D). In the ER-positive/HER2-negative subgroups, the observed AUC score was of 0.949 (95% CI: 0.901; 0.996) (Supplementary Fig. 1A-B).

### Assessment of the DeepGrade classification performance compared to the clinical grade on the resected tumour specimen

**Fig. 4:**
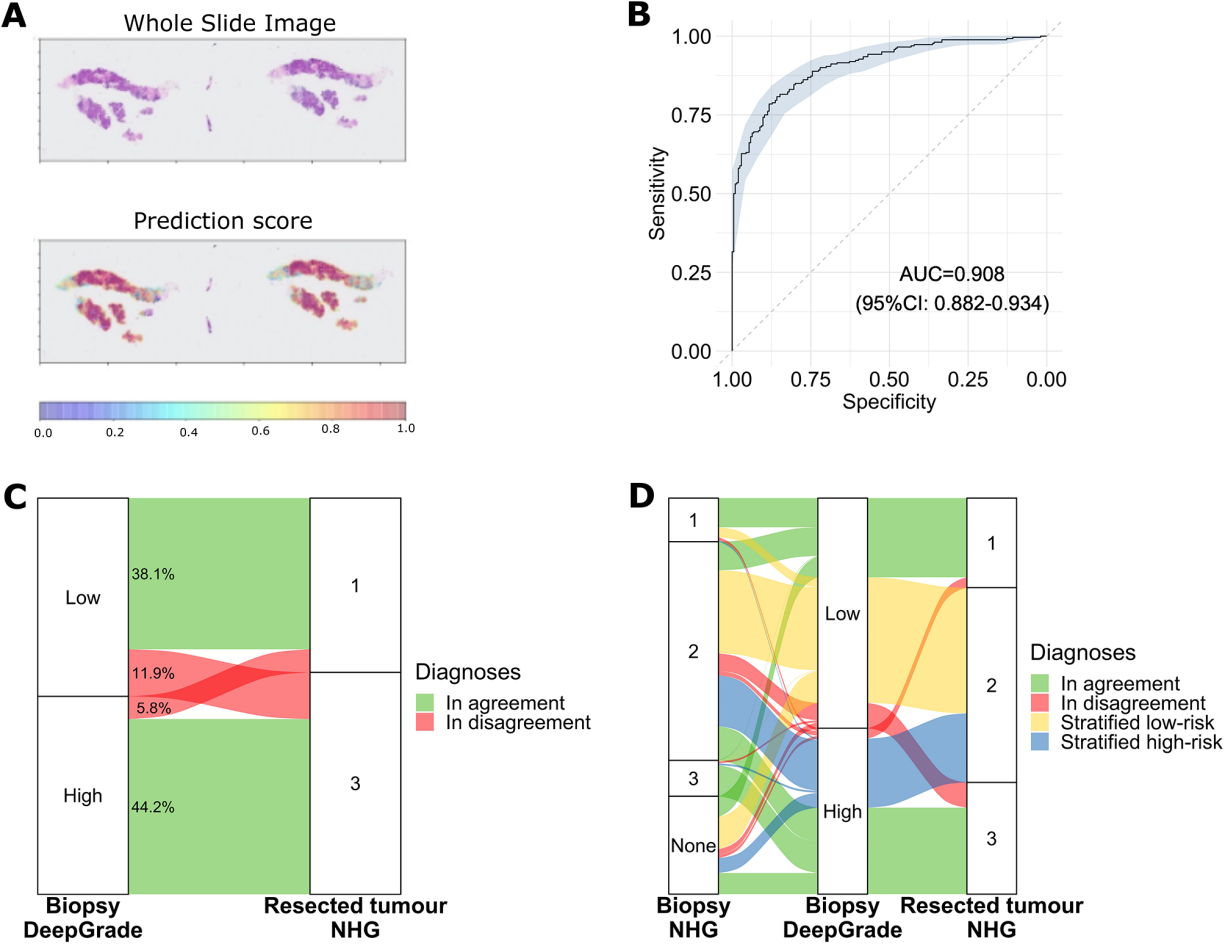
DeepGrade prediction results obtained on biopsy specimens compared to the clinical Nottingham Histological Grade (NHG) assigned by the pathologist on the resected specimen. **A.** Example of a whole slide image with prediction results. Red is more likely to be predicted as high risk, or in other words the predicted probability for each tile to be classified as high-risk. The patient is classified DeepGrade high as the upper percentile of the mean values across all tiles was over the obtained threshold of 0.83. **B.** Receiver Operating Curve (ROC) of the patient-level DeepGrade model prediction versus the resected tumour grades NHG1 and NHG3 assigned by a pathologist. **C.** Sankey plot of the proportion of patients predicted with DeepGrade-high and -low versus the resected tumour grade NHG1 and NHG3. **D.** Sankey plot with results of all biopsy specimen comparing the obtained DeepGrade risk group with both the biopsy NHG and the resected tumour NHG. Diagnoses were in agreement when patients were low-risk biopsy DeepGrade and resected tumour NHG1 or high-risk biopsy DeepGrade and resected tumour NHG3. Patients with resected tumour NHG2 were stratified as either low-risk or high-risk

To test our hypothesis that not only can the DeepGrade model predict the NHG of the biopsy, but also predict the clinically assigned NHG1 and NHG3 grades assigned on the resected specimens, we compared the prediction results obtained (Fig. 4). An example of the DeepGrade prediction results on a biopsy WSI is illustrated in Fig. 4A. We observed an AUC score of 0.908 (95% CI: 0.882; 0.934) when comparing the DeepGrade model prediction obtained on the biopsy versus the clinically assigned NHG1 and NHG3 on the resected tumour (Fig. 4B). Agreement between the risk-group predictions obtained using the biopsy and that of the pathologist for resected specimens with NHG1 and NHG3 was observed for 382 out of 464 patients (82.3%) and the kappa value was 0.65 indicating substantial agreement (Fig. 4C). Sensitivity was of 78.8% and specificity was of 86.8%. When looking at the patients who were in the DeepGrade-low risk group, but who had a resected tumour of NHG3, the clinical biopsy grade was either NHG2 or not graded in 91% of the cases, and only five patients had NHG3 on both biopsy and resected tumour specimen (Fig. 4D). Out of the 432 patients who had a resected tumour with NHG2, 281 (65.0%) were assigned to the DeepGrade-low risk group while 151 (35.0%) were assigned to the DeepGrade-high risk group from biopsies (Fig. 4D). In the ER-positive/HER2-negative subgroup the obtained AUC was of 0.881 (95% CI: 0.846–0.917) (Supplementary Fig. 1C-D). The sensitivity in this subgroup was of 81.3% and the specificity was of 80.6%.

### Comparison between DeepGrade risk groups on biopsy and resected tumour specimens

**Fig. 5:**
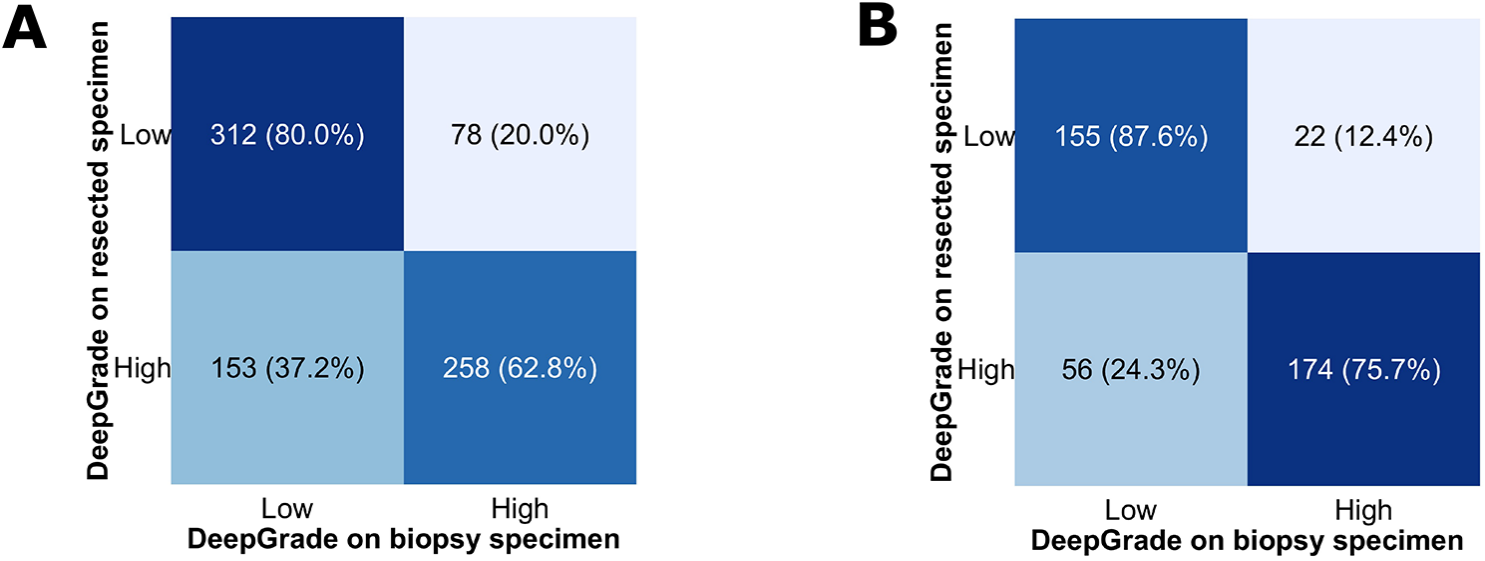
Comparison of DeepGrade risk groups on biopsy and resected specimens. **A.** Confusion matrix for all Nottingham Histological Grade (NHG) grades combined. **B.** Confusion matrix for only resected grades NHG1 and NHG3

To verify whether the results obtained on the biopsy specimens were in line with those obtained on the resected tumour specimen, we compared the DeepGrade risk group for 801 patients for which we had both specimens available (Fig. 5). Almost three quarters of the patients were assigned the same DeepGrade risk group on the biopsy and resected specimens. This proportion was even higher when considering only patients with a resected tumour of NHG1 or NHG3, with 80.8% of the patients assigned the same DeepGrade risk group.

### Prognostic performance of DeepGrade on tumour biopsies

**Fig. 6:**
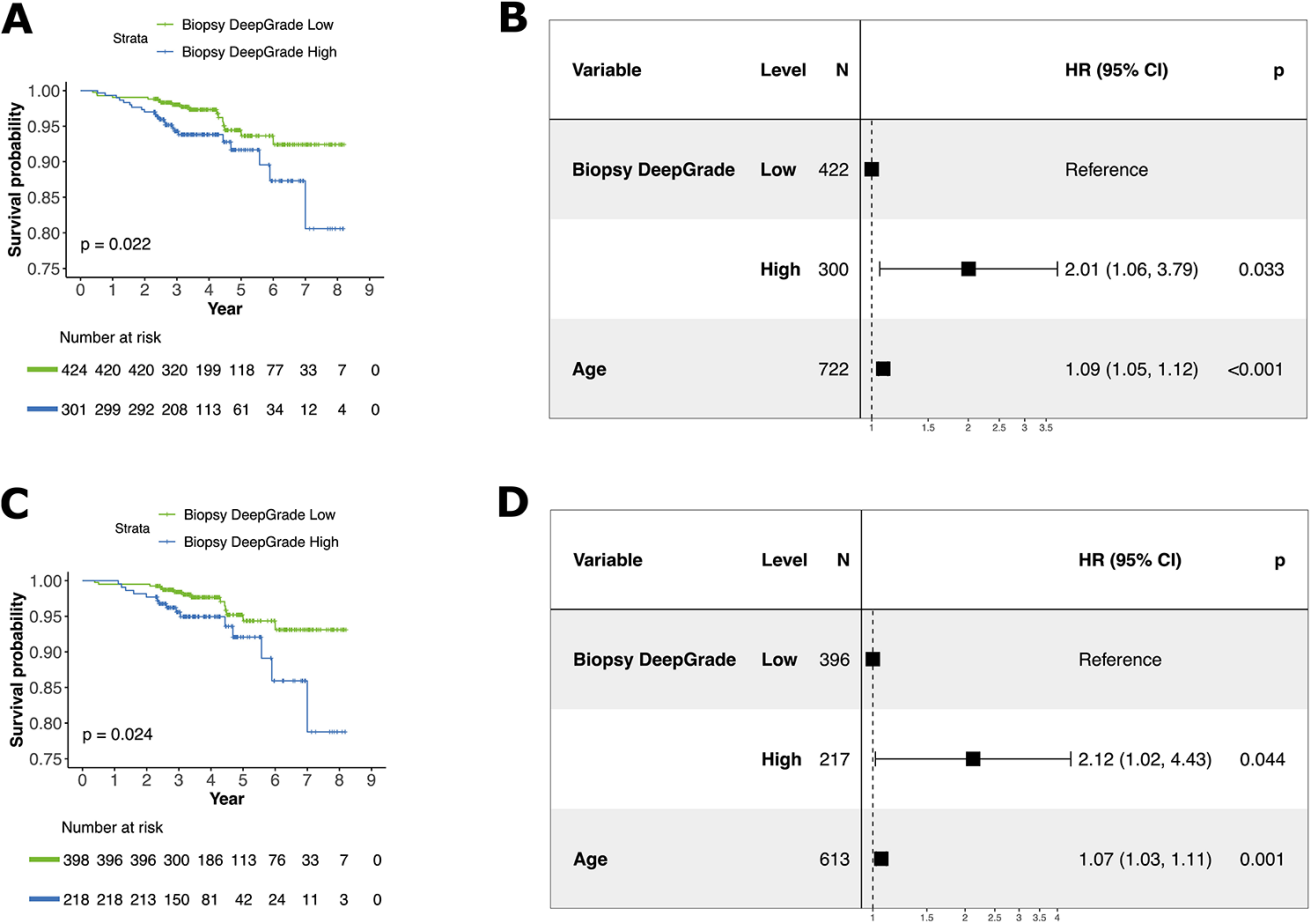
Recurrence-free survival outcomes for breast cancer patients by DeepGrade risk-group obtained on the biopsy specimen. **A.** Kaplan-Meier curves for patients stratified by biopsy DeepGrade-low and -high risk groups. The high-risk group had the worst prognosis. **B.** Forest plot from multivariable Cox proportional hazard regression including the biopsy DeepGrade risk groups and age at diagnosis. Three patients had missing data for age. **C.** Kaplan-Meier curves for ER-positive/HER2-negative patients stratified by biopsy DeepGrade-low and -high risk groups. **D.** Forest plot from multivariable Cox proportional hazard regression for ER-positive/HER2-negative patients including the biopsy DeepGrade risk groups and age at diagnosis

The prognostic performance of the DeepGrade model on biopsy specimens was measured based on recurrence-free survival and was visualised using Kaplan-Meier curves. The independent prognostic value was measured using multivariable Cox proportional hazards model adjusting for age, resembling information available at the biopsy stage. When including all patients, the biopsy DeepGrade model was found to be a predictor of recurrence-free survival with an estimated hazard ratio of 2.01 (*p* = 0.033, 95% CI: 1.06; 3.79) for patients in the DeepGrade-high group compared to those in the DeepGrade-low group, independently of the patient’s age (Fig. 6A-B). Subgroup analyses on patients with ER+/HER2- tumours also showed that DeepGrade was a predictor of recurrence-free survival with an estimated hazard ratio of 2.12 (*p* = 0.044, 95% CI: 1.02; 4.43) for patients in the DeepGrade-high group compared to those in the DeepGrade-low group, independently of the patient’s age (Fig. 6C-D).

## Discussion

The aim of this study was to investigate if the DeepGrade model, previously developed to risk-stratify patients based on resected tumour specimens, could also be used to risk-stratify biopsy specimens. We observed a high classification performance when comparing the DeepGrade predictions on the biopsy specimen to the pathologist-assigned biopsy NHG. Most interestingly, the DeepGrade model could predict the histological grade of the resected tumour specimen while analysing only biopsy material. Furthermore, classification of patients using the DeepGrade model was predictive of recurrence-free survival at point of biopsy.

Neoadjuvant therapy is currently recommended to most HER2-positive and triple negative breast cancers, of which the vast majority are high-grade tumours. The identification of patients with high-grade tumours at the time of biopsy is essential for the decision to treat a patient with neoadjuvant chemotherapy [7, 34], and especially within the larger ER-positive, HER2-negative subgroup [7]. However, conventional histological grading of biopsies by pathologists remains challenging and most biopsies are assigned the intermediate NHG2, or are not graded at all [14, 18]. This lack of precision in biopsy grading leads to a discrepancy between pathologists, and in one cohort up to 45% of women had a change in diagnosis between the biopsy and the resected tumour [18]. We found that 41% of patients who were not assigned a grade on the biopsy were assigned to the high-risk group by DeepGrade, of which 54% were actually assigned as NHG3 by pathologists on their resected tumour specimen. Earlier diagnosis could assist with earlier treatment decisions.

Several studies have developed models to predict grade using deep learning models on WSI from resected tumour specimens but not using core needle biopsies [20–23, 35, 36]. In particular, Wang et al. obtained an AUC of 0.907 for DeepGrade in their external data regarding resected tumours which is in line with the accuracy we obtained on the biopsy specimen when comparing to resected tumours of NHG1 versus NHG3 (0.908) [23]. Others who have predicted grade into two groups (low-grade and high-grade) on resected tumour specimens obtained agreements around 80%, and kappa values between 0.59 and 0.64 [35, 37]. Despite predicting the resected specimen grade using only biopsy material, we achieved high performance results among NHG1 and NHG3 tumours with an agreement of 82% and a kappa value of 0.65 between biopsy DeepGrade risk groups and pathologist-assigned NHG on resected tumours. As a comparison, only 32% of the 463 cases who were NHG1 or NHG3 on the resected specimen were also assigned NHG1 or NHG3 on the biopsy specimen by a pathologist. In the literature, agreement between biopsy and resected tumour NHG by pathologists for all three grades is usually around 75% and ranges from 59–91% [38]. The results presented were obtained without performing prior tumour predictions as the biopsy material is smaller and the presence of benign tissue should not influence significantly the presence of high risk morphological patterns that are identified by the DeepGrade model.

The use of biopsy specimens in computational pathology within breast cancer is relatively rare in the literature, as opposed to the work performed in prostate cancer [39–41]. A number of studies focused on the identification of tumour areas [42–45], while others aimed to predict the response to neoadjuvant therapy, in part using grade as their training material [46, 47]. The DeepGrade model extracts histological grade-related morphological information from images using deep CNN models. To date no risk stratification methods for survival prediction have been proposed using biopsy material, however different approaches have been suggested related to grade [20], grading sub-components [22, 48], and intra-tumour heterogeneity [49] using resected tumour specimens only. The proposed methodology in this study could be used as a decision support tool to complement pathologists and treating physicians, as it establishes a risk assessment of all tumours, including those that are hard to grade. It also has the benefit of providing a solution that is less costly and with shorter waiting times, both for the patient and for the healthcare providers than other methods used for risk stratification such as Oncotype DX (Exact Sciences Corp., Madison, WI, USA) or Prosigna (Veracyte Inc., South San Francisco, CA, USA) gene expression assays [50, 51]. Both have been developed for patient risk stratification and treatment decisions on the resected tumour specimens, but have also been applied outside of their intended use, for assessment of core needle biopsy specimens with conclusive results [52–55].

This is the first study demonstrating risk stratification of NHG2 tumours already at the time of biopsy using deep learning. Although several methods are available and implemented in clinical routine to risk stratify patients, most use gene expression profiling assays [52–55], which are time-consuming methods and remain costly [56]. The risk stratification method presented in this study has the advantages of providing a result to the pathologist in a short time-frame and at a very low cost given most pathology laboratories in high-income countries already use digitised WSI to some extent in routine diagnostics [23].

Limitations of this study include the fact that the study was based on retrospective material in order to obtain a large enough sample size when only including one hospital. Nonetheless, the small number of recurrence events leads to low-powered survival analyses. Even though most pathologists would not have direct access to the biopsy grade when assigning a grade to the resected specimen, it was possible for them to look into the patient’s electronic record and to make a decision based on the previously assigned grade. The discrepancies in diagnoses observed here as well as in previous work would however point into the direction that the two grades are given independently. Furthermore, a limitation of the present study is that a subset of this study, 173 patients (19.3%) were included as training data of the initial DeepGrade model [23]. However, the biopsy material itself was never used for the training of the original DeepGrade model representing in itself a fully independent set from the original data, and results presented in Supplementary Fig. 2 show that performance remains high. In the future, further analyses on patients from another hospital would be beneficial to confirm the results obtained.

## Conclusions

In conclusion, we found that the resected tumour grade could be predicted by DeepGrade based on using only biopsy specimens. With relatively simple implementation, high-risk tumours could therefore be identified at the preoperative stage. Like in resected tumours, DeepGrade could also stratify NHG2 tumours on biopsy specimens into low- and high-risk groups. In the future, this could provide decision support to pathologists as well as treating physicians to improve the quality of relevant information for clinical decisions earlier on in the process, and thus potentially reduce both over- and under-treatment of patients in the neoadjuvant setting.

### Electronic supplementary material

Below is the link to the electronic supplementary material.

Supplementary Material 1

## Data Availability

The datasets analysed during the current study are not publicly available due to local privacy laws but are available from the corresponding author upon reasonable request.
