# Supplementary material for: Deep learning-based risk stratification of preoperative breast biopsies using digital whole slide images
Source: Breast Cancer Res. 2024 Jun 3;26:90. doi: 10.1186/s13058-024-01840-7 (PMC11145850; doi:10.1186/s13058-024-01840-7)
Supplement: Supplementary file 1 — Supplementary Material 1 [file 13058_2024_1840_MOESM1_ESM.pdf]

Supplementary Figures

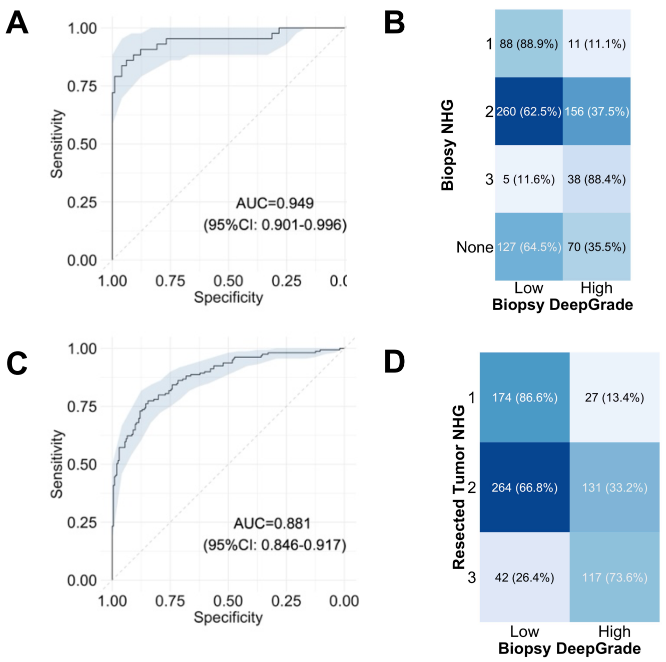

Supplementary Figure 1. Performance results including only ER-positive/HER2-negative patients (n=755). A. Receiver Operating Curve (ROC) of the patient-level prediction obtained by the DeepGrade model compared to biopsy NHG1 and NHG3. B. Confusion matrix for all 755 patients comparing biopsy NHG and predicted DeepGrade risk group. B. Receiver Operating Curve (ROC) of the patient-level prediction obtained by the DeepGrade model compared to the resected tumour NHG1 and NHG3. D. Confusion matrix comparing the resected tumour NHG and predicted DeepGrade risk group.

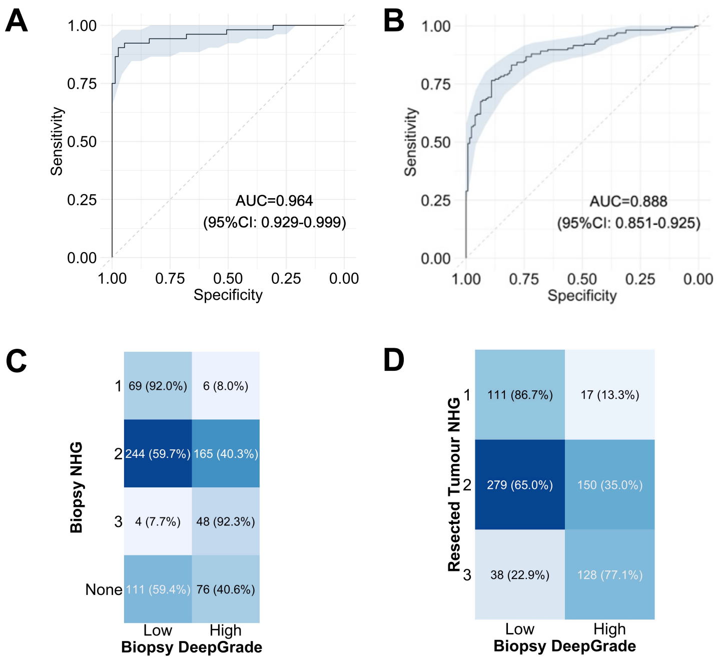

Supplementary Figure 2. Performance results including only cases for whom the resected tumour was not used as part of the training set of the initial DeepGrade model (n=723). A. Receiver Operating Curve (ROC) of the patient-level prediction obtained by the DeepGrade model compared to biopsy NHG1 and NHG3. B. Receiver Operating Curve (ROC) of the patient-level prediction obtained by the DeepGrade model compared to the resected tumour NHG1 and NHG3. C. Confusion matrix for all 723 patients comparing biopsy NHG and predicted DeepGrade risk group. D. Confusion matrix comparing the resected tumour NHG and predicted DeepGrade risk group.
